# Drought resistance of tobacco overexpressing the *AfNAC1* gene of *Amorpha fruticosa* Linn.

**DOI:** 10.3389/fpls.2022.980171

**Published:** 2022-08-16

**Authors:** Minghui Li, Ziang Liu, Chenxi Liu, Fengjin Zhu, Kai Wang, Zhenyu Wang, XiuFeng Li, Xingguo Lan, Qingjie Guan

**Affiliations:** ^1^Key Laboratory of the Ministry of Education for Ecological Restoration of Saline Vegetation, College of Life Sciences, Northeast Forestry University, Harbin, China; ^2^College of Forestry, Northeastern Forestry University, Harbin, China; ^3^Agriculture and Rural Bureau, Suihua, China; ^4^Key Laboratory of Molecular Design Breeding of Soybean, Northeast Institute of Geography and Agroecology, Chinese Academy of Sciences, Harbin, China

**Keywords:** *Amorpha fruticosa* Linn., WGCNA, NAC transcription factor, drought stress, tobacco

## Abstract

Plants are often adversely affected by abiotic stresses such as drought, low temperature, and salinity during growth, and plant NAC-like transcription factors are involved in regulating growth and developmental processes in response to stresses such as drought and salinity. In this study, to investigate the function of *AfNAC1*, a co-expression network of *AfNAC1* genes was constructed using gene expression data from the Chinese legume deciduous shrub, *Amorpha fruticosa* Linn. A 576 bp NAC transcription factor (*AfNAC1* gene, MN180266) encoding 191 amino acids was isolated from *Amorpha fruticosa* seedlings by RT-PCR. qRT-PCR showed that the *AfNAC1* gene was expressed in the roots, stems, leaves, and flowers of *Amorpha fruticosa.* However, drought stress significantly increased root expression, and the AfNAC1 protein was localized in the nucleus by green fluorescence detection. This study analyzed the drought resistance of overexpressing tobacco in depth. Under natural drought stress, the chlorophyll and antioxidant enzyme activities of overexpressing plants were significantly higher than those of wild-type (WT) plants, but the MDA content was lower than that of WT; after rehydration the Fv/Fm values of *AfNAC1*-overexpressing tobacco recovered faster than those of wild-type tobacco and rapidly reached the control levels; *AfNAC1* may be involved in the regulation of the photosystem and indirectly in the regulation of the plant in response to drought stress.

## Introduction

Drought is one of the most important abiotic stresses that limit plant growth, development, and productivity, and drought stress severely limits plant growth and crop yield. Therefore, reducing the effects of drought injury is of considerable agro-pastoral importance (Harris et al., 2007; Martínez et al., 2007; Rivero et al., 2007; Degenkolbe et al., 2009) The drought resistance of plants has been studied for many years, and current studies have shown that species with increased drought resistance include *buckthorn*, *crested hazel*, *tiger hazel*, and *Amorpha fruticosa* (Wang, 2003; Hou et al., 2009; Gao, 2018), and the major silvicultural species of the Loess Plateau: *oleander*, *cypress,* and *acacia* (Liu et al., 2003). NAC-like transcription factors are one of the largest families of transcription factors specific to plants, and they play an important role in response to various abiotic stresses, such as salt, drought, and damage. In herbaceous plants such as *Arabidopsis thaliana* (Mahmood et al., 2019), rice (Li, 2014), alfalfa (Shen et al., 2014), and land cotton (Shah, 2013), NAC transcription factors were found to be upregulated in response to abiotic stresses, and in woody plants such as tamarisk (Lu et al., 2019) and poplar (Wang et al., 2015), PEG-6000 mimics drought stress; the transcriptome detected upregulation of NAC-like transcription factors in response to drought-induced differences in gene expression (Sun et al., 2021). The NAC family has been found to function in a variety of processes, including apical meristem organization (Takada et al., 2001), flower development (Sablowski and Meyerowitz, 1998), cell division (Kim et al., 2006), leaf senescence (Breeze et al., 2011), secondary wall formation (Zhong et al., 2010), and biotic and abiotic stress responses (Olsen et al., 2005; Christianson et al., 2010; Tran et al., 2010; Nakashima et al., 2012). To date, intensive studies in model plants such as Arabidopsis and rice have revealed that a typical NAC protein contains a highly conserved N-terminal DNA-binding NAC structural domain and a variable C-terminal transcriptional regulatory region. The NAC structural domain containing 150–160 amino acids is divided into five sub-structural domains. Three of these sub-structural domains, A, C, and D, are highly conserved across species, whereas the other two sub-structural domains, B and E, are relatively less conserved (Ooka et al., 2003). The function of the NAC structural domain is related to nuclear localization and DNA binding, forming homodimers or heterodimers with other NAC structural domain-containing proteins (Olsen et al., 2005). In contrast, the highly differentiated C-terminal region functions as a transcriptional regulatory region, acting as a transcriptional activator or repressor, but it often has simple amino acid repeats and regions rich in serine and threonine, proline and glutamine, or acidic residues (Olsen et al., 2005; Puranik et al., 2012). Some NAC TFs also contain transmembrane patterns in the C-terminal region responsible for anchoring the plasma membrane or endoplasmic reticulum, and these NAC TFs are membrane-associated and are referred to as NTLs (Seo et al., 2008; Seo and Park, 2010). The drought tolerance function of NAC-like transcription factors in *A. fruticosa* has not been studied in sufficient depth.

*Amorpha fruticosa* Linn. is a perennial, leguminous, easily propagated shrub of the genus *Amorpha* with less stringent soil requirements and many favorable characteristics such as tolerance to cold, infertility, flood, drought, and salt (Kim et al., 2011; Yin et al., 2014; Fan et al., 2017). In this study, the *AfNAC1* gene was cloned from the leaf transcriptome sequence of *A. fruticosa* by RT-PCR, and the specificity of *AfNAC1* gene expression was examined by bioinformatics analysis; validation experiments were conducted for the prediction of bioinformatics subcellular localization of the *AfNAC1* gene; drought tolerance of overexpressed tobacco plants at the germination and seedling stages was analyzed. The molecular mechanism of the *AfNAC1* transcription factor that is indirectly or directly involved in the photosystem II response of plants to enhance the regulation of drought stress was investigated; the findings provide a theoretical basis for studying the drought resistance function of NAC transcription factors in woody plants.

## Materials and methods

### Plant material

Seeds of *A. fruticosa* were collected from the green belt of Songyuan East Highway, Ningjiang District, Songyuan City, Jilin Province, China. The seeds of *Nicotiana benthamiana* were kept by this laboratory, the Key Laboratory of the Ministry of Education for Restoration and Reconstruction of Saline Land Vegetation, Northeast Forestry University (NEFU).

### Strain, vector, and reagents

*E. coli* JM109 and *Agrobacterium* EHA105 were stored in our laboratory. *pMD18-T* vector was purchased from TaKaRa, and the *pBI121-MCS-GFP* plant expression vector was stored in our laboratory. T4-DNA ligase and Ex-Taq DNA polymerase were purchased from TaKaRa. The real-time quantitative fluorescent dye SYBR Green qPCR Master Mix was purchased from Gel Extraction Kit; acetosyringone was purchased from Solarbio Biologicals. Other reagents were made in China with analytical purity.

### Co-expression network analysis of *AfNAC1* gene

The seeds were surface disinfected with 75% alcohol and 5% sodium hypochlorite for 5 min and then rinsed three times with distilled water. The seeds were sown in fine sand and placed in an incubation chamber with water. Four-week-sized seedlings of *A. fruticosa* were grown hydroponically in a 20% PEG 6000 (polyethylene glycol 6,000) solution for 72 h. Whole seedlings were sampled separately from the stressed and unstressed treatments, rapidly frozen in liquid nitrogen, and stored in a − 80°C refrigerator. After transcriptome sequencing results were obtained (Sun et al., 2021), co-expression networks of genes were constructed using the WGCNA (Weighted Gene Co-expression Network Analysis) R package.

### Cloning and bioinformatics analysis of the *AfNAC1* gene in *Amorpha fruticosa* Linn.

The sequence of the upregulated *NAC1* gene (c169215.graph_c0) of *A. fruticosa* under drought stress was analyzed by transcriptome sequencing. Sequencing data can be found in the Big Sub database^1^ under the login number CRA002113. The online website NCBI was used to find the ORF and code amino acids of *AfNAC1*, among others. Specific primers (see Supplementary Table S1 for AfNAC1-F1/R2) were designed using Premier 5.0 software, and the ORF fragment was amplified by RT-PCR using cDNA from *A. fruticosa* seedlings as a template, and the product was ligated to the pMD18-T vector. Various physicochemical properties of the proteins were analyzed using EXPASy; the protein structures were predicted using online software.^2^ An amino acid sequence homology match was performed using DNAMAN, and a phylogenetic tree was constructed using MEGA6.

### Expression characteristics of *AfNAC1* gene in shoots under drought stress

Total RNA was extracted from the roots, stems, leaves, and flowers of *A. fruticosa*, and seedlings were treated with different concentrations of PEG-6000 (0, 10, 20, and 30% w/v groups) for 72 h. All aboveground seedlings (stems and leaves) of all groups were sampled for total RNA extraction. Total RNA was extracted from leaves and roots of seedlings treated with 20% PEG for 0, 6, 12, 24, and 48 h, separately. cDNA was obtained by reverse transcription and used as a template to design the internal reference and gene quantification primers (qRT-PCR-F3/R4; see Supplementary Table S1) for qPCR reactions. The data were collected using an MxPro-Mx3000P mechanical system, then analyzed using a DPS data processing system and plotted using Origin software.

### Subcellular localization analysis of *AfNAC1*

The specific primers (*AfNAC1-GFP*-F5/R6 in Supplementary Table S1) were designed by introducing XbalI and SalI digestion sites, and the recombinant plasmid pBI121-*AfNAC1-GFP* (a binary plant expression vector under CaMV 35S initiation) was constructed by double digestion after PCR amplification using this primer. *Agrobacterium tumefaciens* EHA105 containing the pBI121-*AfNAC1-GFP* recombinant plasmid was selected for injection into fresh tobacco leaves, and the injected-infested tobacco was incubated in the dark for 12–16 h. After incubation under normal light for 3 days, fluorescence and DAPI staining for subcellular localization were observed under confocal microscopy (Tian et al., 2021).

### Genetic transformation and resistance analysis of tobacco overexpressing *AfNAC1*

#### Acquisition of transgenic tobacco

Tobacco leaves were infested with recombinant *Agrobacterium tumefaciens* EHA105 containing pBI121-*AfNAC1-GFP* plasmid DNA and cultured on MS-As medium for 3 days. Shoot differentiation was induced on tobacco screening differentiation medium at 50 mg/l Kana (Kanamycin), and rooting was further induced in rooting medium (1/2MS + 50 mg/l Kana +250 mg/l Carbenicillin). Seeds of the T1 generation were harvested in pot culture (Cui et al., 2006), and seeds of the T3 generation were harvested by Kana screening sowing transplantation.

#### Analysis of drought resistance in tobacco overexpressing *AfNAC1*

Transgenic and wild-type tobacco plants were subjected to qRT-PCR analysis to detect relative expression. T3-generation tobacco seeds were spotted in MS medium at various mannitol concentrations (0, 200, 250, and 300 mm) to simulate drought; 20 days after germination, the germination rate and fresh weight were measured; and the plants were tested for MDA content, SOD enzyme activity, and chlorophyll content, and analyzed for changes in root length in each strain. WT and overexpression (T3-#1, #2, #5) seeds were sown in soil and incubated at 25°C under 8/16 h light/dark conditions for 4 weeks followed by 15 days’ drought treatment and 3 days’ rehydration treatment. Morphological observations were performed and photographed before treatment, at 15 days of drought and at 3 days of rehydration, and the photosynthetic capacity, Fv/Fm values of drought-rehydrated leaves were measured with a FluorCam open chlorophyll fluorescence imaging system (Miura et al., 2007). Statistical analysis of the transgenic and control lines was conducted using Prism9, and a DPS data processing system was used to analyze the significance of one-way differences.

WT and overexpressed (T3-#1, #2, and #5) tobacco seeds were sown in soil and incubated for 4 weeks at 25°C under 8/16 h light/dark conditions， with drought treatment as the experimental group and watered roots as the control group. Both the DAB staining method and the NBT staining method have been described in previous reports (Guan et al., 2015, 2018). The experimental and control groups were sampled, RNA was extracted, and NtActin was used as an internal reference to analyze the expression levels of oxidative stress genes. The levels of chlorophyll (Qiu et al., 2016), proline (Ryu et al., 2014), superoxide dismutase (SOD; Kang et al., 2016), and peroxidase (POD; Wang et al., 2018) were also measured.

Statistical analysis of transgenic and control lines was performed using Prism9 and the significance of ANOVA was analyzed using the DPS data processing system. Data are expressed as mean ± SE with three biological replicates. Student’s *t-*test was performed at *p* < 0.05 to analyze significant differences.

## Results

### Gene co-expression network analysis

**Figure 1:**
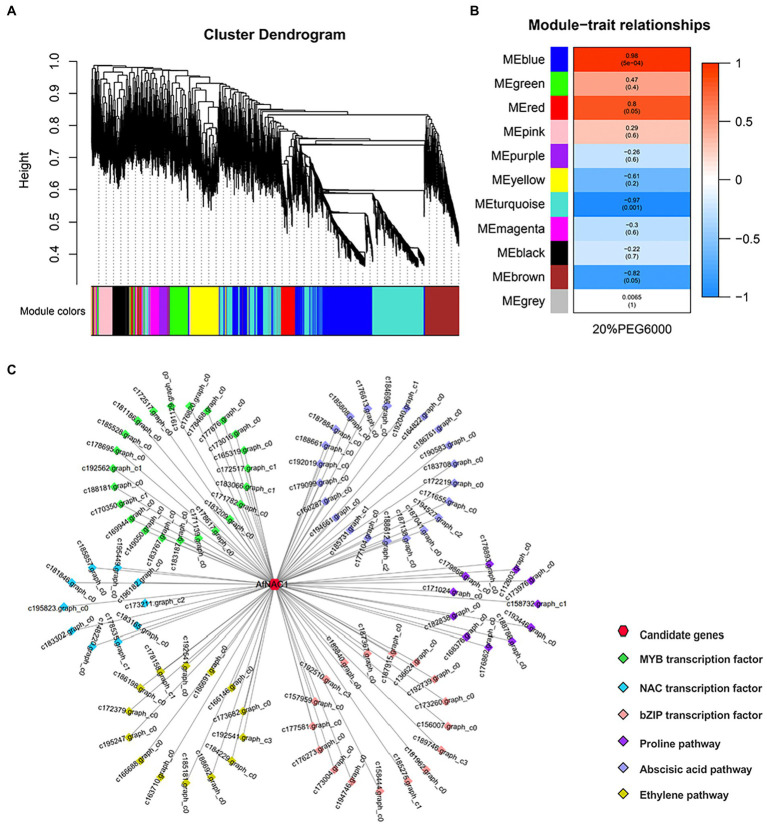
Results of gene co-expression analysis. **(A)** Gene clustering map. The different colors indicate the constructed vector complex modules, the phylogenetic tree indicates the hierarchical clustering of different samples, and each sample corresponds to one color of the corresponding module **(B)** Heat map of co-expression module associated with 20% PEG6000. **(C)** Correlation analysis between genes.

After screening the transcriptome sequencing results, 6,979 genes were obtained to construct a co-expression network, yielding a total of 11 modules (Figure 1). The *AfNAC1* gene was found in the “blue,” “MEred,” and “pink” modules. The “blue” module showed a significant positive correlation (*r*^2^ = 0.98, value of *p* = 5 × 10^−4^). It was also found that major types of gene clusters, including ABA assimilation classes and proline assimilation classes, were directly correlated with the *AfNAC1* co-expression network in the local co-expression network map. This suggests that *AfNAC1* transcription factors are involved in the regulation of drought resistance in plants.

### Cloning and bioinformatics analysis of the *AfNAC1* gene of *Amorpha fruticosa* Linn.

**Figure 2:**
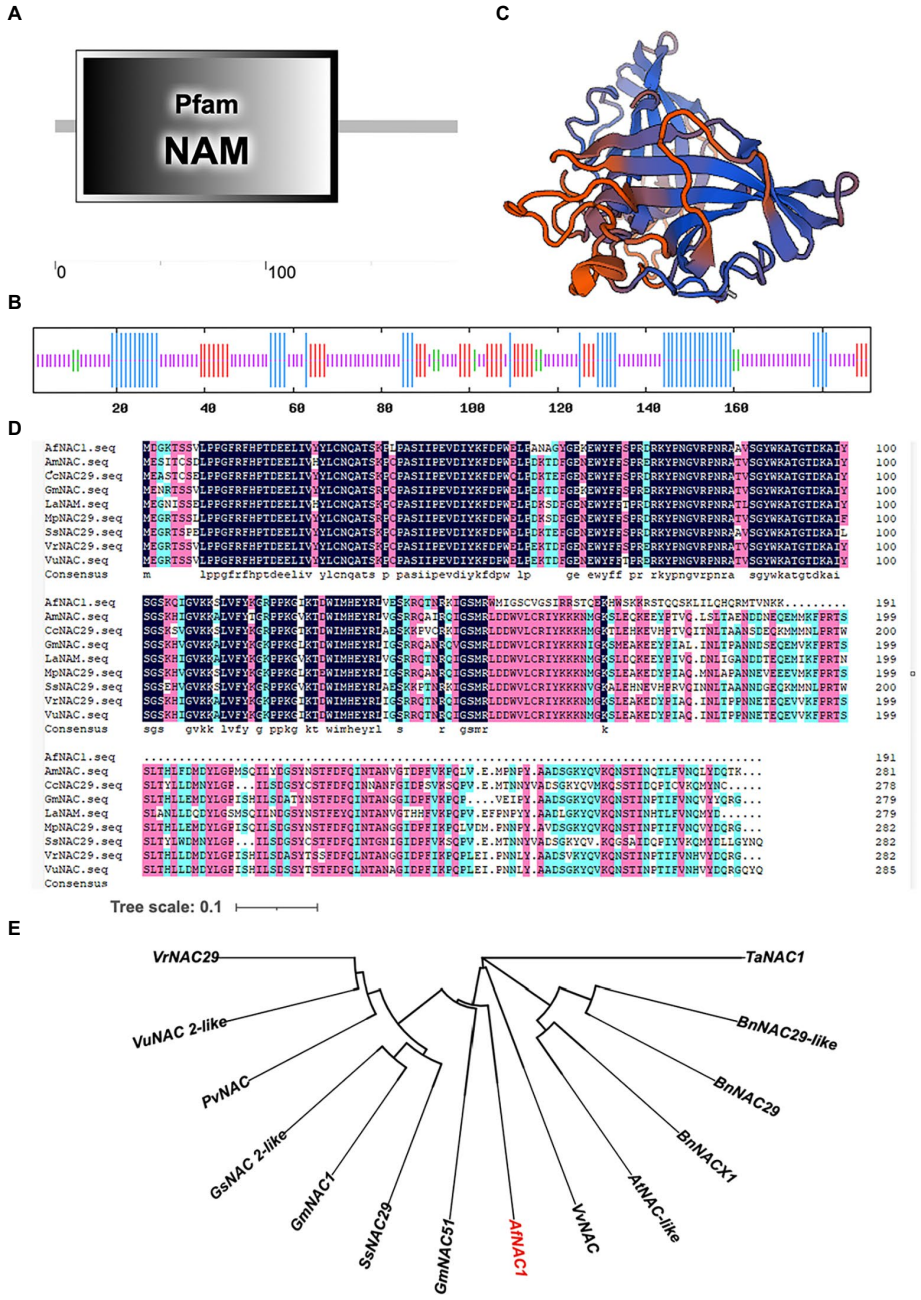
Results of bioinformatics analysis of the *AfNAC1* gene. **(A)** Primary structure of the protein, including the NAM structural domain (1–100 aa). **(B)** Secondary structure, where the blue color indicates α-helix, the green color indicates β-steering, the red color indicates extended chain, and the purple color indicates irregular coiling. **(C)** Tertiary structure. **(D)** Results of multiple comparisons of amino acid sequences of NAC transcription factors with other different species. **(E)** Phylogenetic evolutionary tree. Scale bar indicates 0.1 amino acid substitution per site.

The transcriptome *AfNAC1* gene (c169215.graph_c0) was analyzed for its full length of 1887 bp, of which the non-translation area at the 5′ and 3′ ends are 498 and 813 bp, respectively. The CDS fragment ORF was obtained by RT-PCR and ligated to the pMD18-T vector. Sequencing and analysis showed that the ORF is 576 bp, encoding 191 amino acids (Supplementary Figure S1); the predictive protein molecular weight is 22.04 kDa. The analysis showed that the primary structure of the AfNAC1 protein consists of an NAC domain (NAM) at the N terminus (Figure 2A). The secondary structure consists of 46 α-helixes (24.08% of the total length), 9 β-turns (4.71%), 32 extended strands (16.75%), and 104 random coils (54.45%; Figure 2B). SWISS-MODEL online analysis tool analyzed the tertiary structure; it was found that the existence of the NAC structure was consistent with the predicted results of the secondary structure (Figure 2C). The amino acid sequences of *AfNAC1* were analyzed by multiple sequence alignment and have the sequence characteristics of the NAC family (shown in Figure 2D). The construction of a phylogenetic tree revealed that AfNAC1 is most closely related to the GmNAC51 protein (Figure 2E).

### Expression characteristics of *AfNAC1* gene under tissue and drought stress

**Figure 3:**
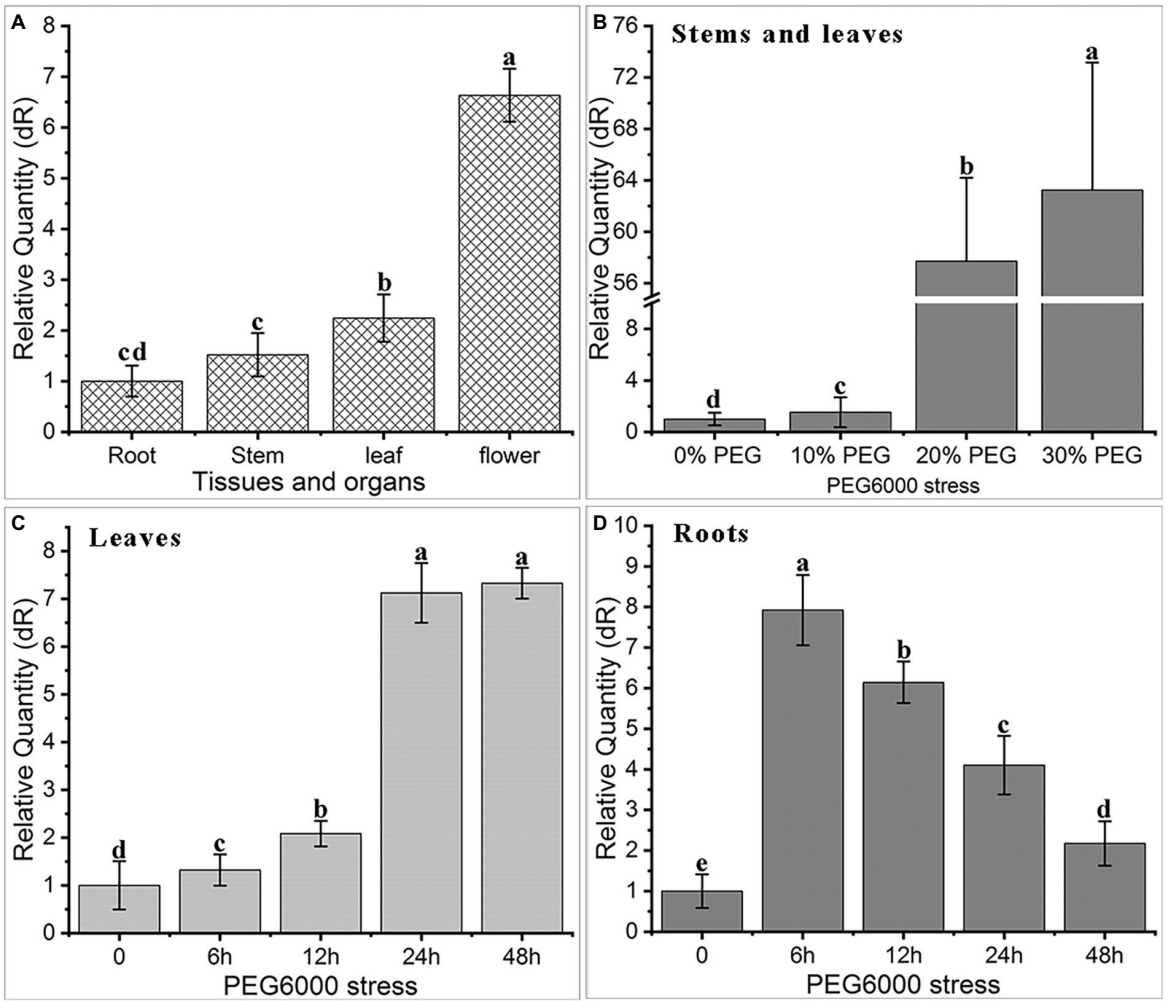
Expression characteristics of *AfNAC1* gene in tissue organs and under PEG6000 stress. **(A)** Tissue organs: root, stem, leaf, and flower; **(B)** 0, 10, 20, and 30% PEG6000 stress; **(C)** leaves under 0, 6, 12, 24, and 48 h PEG6000 stress; **(D)** roots under 0, 6, 12, 24, and 48 h PEG6000 stress; error lines indicate standard error of three biological replicates, significant difference at *p* < 0.05 level.

Quantitative real-time PCR (qRT-PCR) analysis showed that *AfNAC1* is constitutively expressed in different tissues of *A. fruticosa*, including roots, stems, leaves, and flowers, with the highest abundance in flowers and the lowest abundance in the roots (Figure 3A).

We also evaluated the differential expression of the *AfNAC1* gene under PEG6000 simulated drought stress treatment (72 h), and the gene expression level increased significantly with increasing PEG6000 concentration. Compared with the control, the abundance of *AfNAC1* was increased by approximately 55-fold and 64-fold in stems and leaves of *A. fruticosa* under 20 and 30% PEG6000 treatment (Figure 3B), respectively. Under 20% PEG6000 treatment, the highest level was accumulated in leaves at 24 h (Figure 3C), but the highest expression level was reached in roots at 6 h (Figure 3D), and then gradually decreased with the increase in treatment time.

### *AfNAC1* was localized in the nucleus

**Figure 4:**
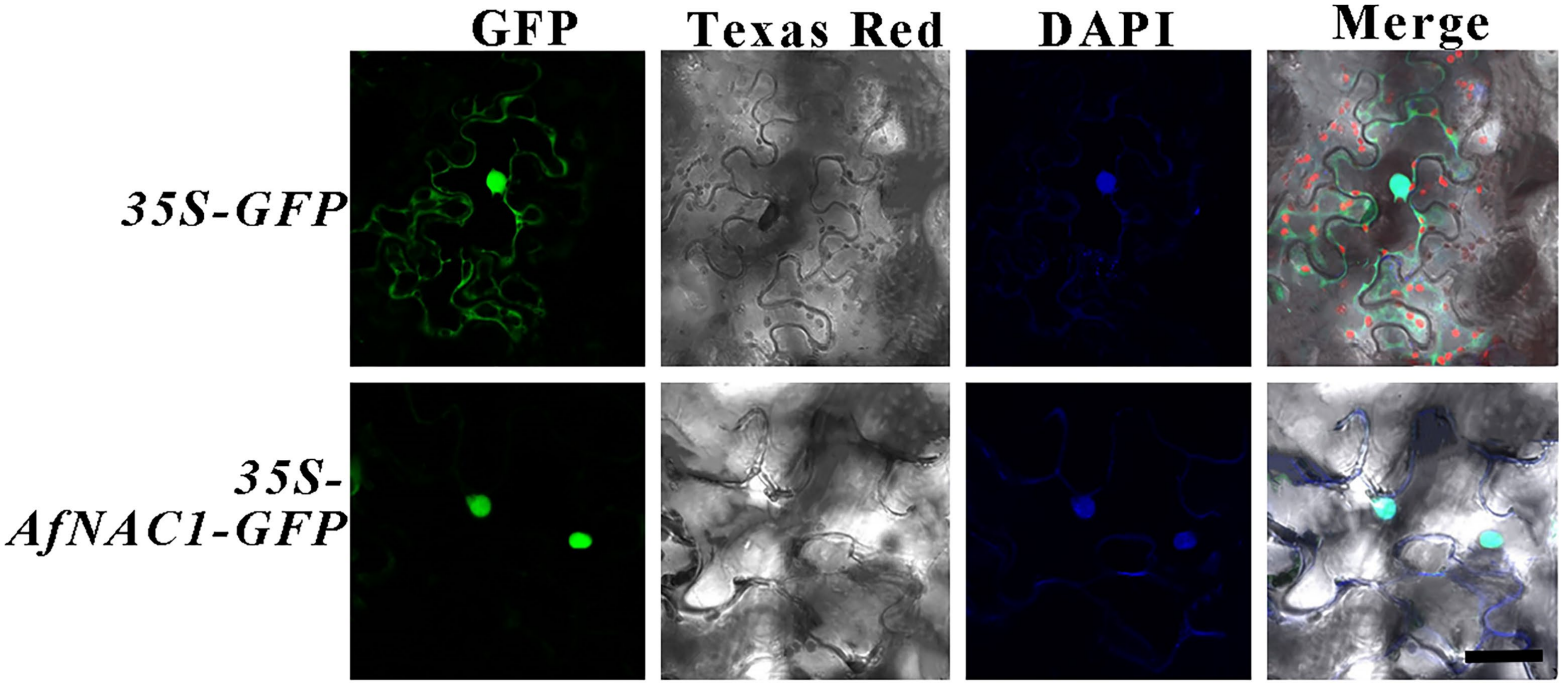
Subcellular localization of AfNAC1 protein in tobacco cells. Bar, 50 μm.

To investigate the subcellular localization of AfNAC1, the green fluorescent protein (*GFP*)-AfNAC1 fusion protein was driven by the *35S* promoter and transiently expressed in *Nicotiana benthamiana* leaves. AfNAC1 localized in the nucleus and was able to overlap with the signal of the nuclear–specific dye DAPI (Figure 4).

### Overexpression of *AfNAC1* transgenic tobacco

To examine whether *AfNAC1* could enhance the drought tolerance of plants, transgenic tobacco was generated. Constructs of the *AfNAC1*-fused *GFP* gene driven by the *35S* promoter were transformed into tobacco, and five independent transgenic lines were identified (Supplementary Figure S2). The expression level of *AfNAC1* transgenic tobaccos was analyzed by quantitative RT-PCR, and the expression of lines one, two, and five was relatively higher than that of others, so these three lines were selected for subsequent studies (Supplementary Figure S2).

### Tolerance of drought stress during germination in tobacco overexpressing *AfNAC1*

**Figure 5:**
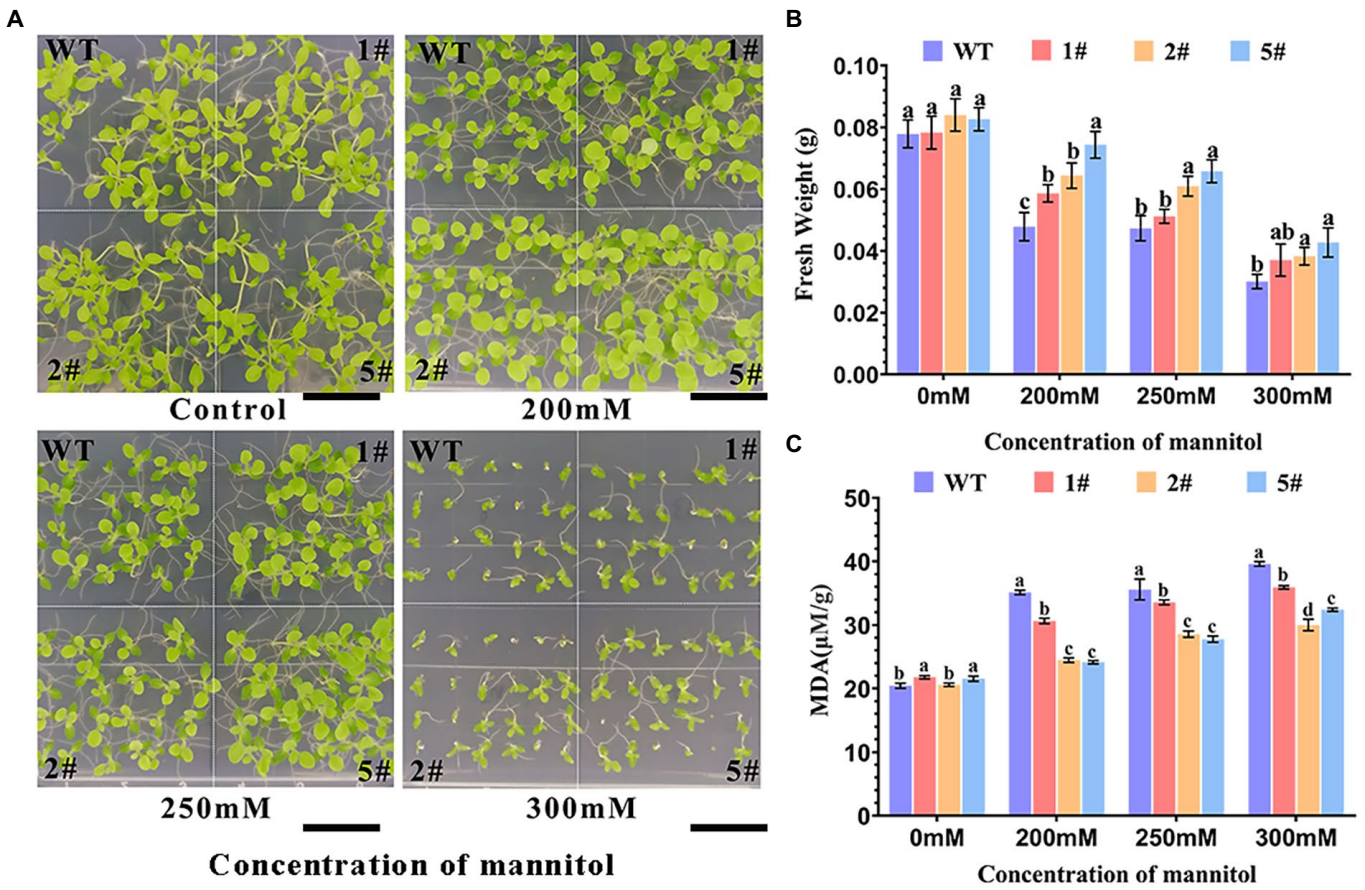
Plant root growth of an *AfNAC1-*overexpression strain and wild-type plants under different concentrations of mannitol stress treatment. WT: wild-type plants; #1, #2, and #5 are three different strains of overexpression plants. **(A)** Plant root elongation phenotype. **(B)** Plant root measurement data. **(C)** Malondialdehyde content measurement data. Error lines indicate the standard errors of three biological replicates with significant differences at the *p* < 0.05 level. Bar, 7.5 mm.

In this study, we analyzed the germination rate of transgenic tobacco under mannitol treatment. The germination rate of wild-type tobacco was significantly inhibited by increasing mannitol concentration than that of transgenic tobacco. After 20 days of incubation, the two pairs of leaves of *AfNAC1* transgenic tobacco were significantly larger than the wild type under 250 mm mannitol treatment, whereas the leaves of transgenic tobacco had significantly more leaves than the wild type under 300 mm mannitol conditions (Figure 5A). The fresh weight of wild-type tobacco decreased significantly with increasing mannitol concentration, while the fresh weight of transgenic tobacco was consistently higher than wild type under mannitol treatment (Figure 5B). More transgenic tobacco was detected with significantly lower malondialdehyde content than wild-type tobacco (Figure 5C). These results suggest that overexpression of the *AfNAC1* gene improves the tolerance of tobacco to mannitol stress during the germination growth period and suggest that *AfNAC1* may play a regulatory role in the drought stress response.

### Tolerance to mannitol mimetic drought stress at the seedling stage

**Figure 6:**
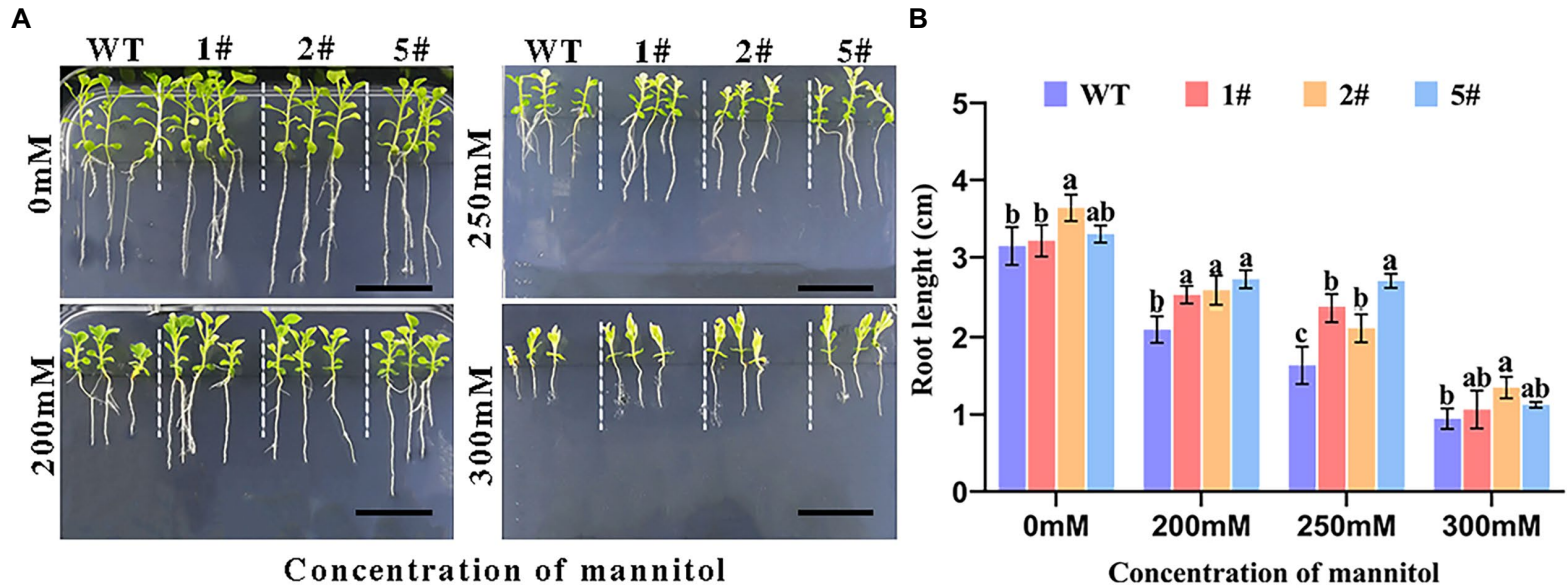
Plant root growth of strain overexpressing *AfNAC1* and wild-type plants under different concentrations of mannitol stress treatment. WT: wild-type plants; #1, #2, and #5 are three different strains of overexpressing plants. **(A)** Plant root elongation phenotype. **(B)** Plant root measurement data. Error lines indicate the standard errors of three biological replicates with significant differences at the *p* < 0.05 level. Bar, 20 mm.

Seedlings at the four-leaf stage with consistent germination growth were pressurized with mannitol and incubated vertically for 20 days (Figure 6A). Wild-type tobacco was more severely inhibited than transgenic tobacco as mannitol concentration increased, and root growth was similarly inhibited. Under mannitol treatment, root length was significantly shorter, whereas the root length of transgenic tobacco was longer than that of wild type, especially under 300 mm mannitol treatment (Figure 6B). The findings suggest that overexpressed *AfNAC1* tobacco has higher drought tolerance during the seedling stage.

### Tolerance of transgenic tobacco to natural drought stress

**Figure 7:**
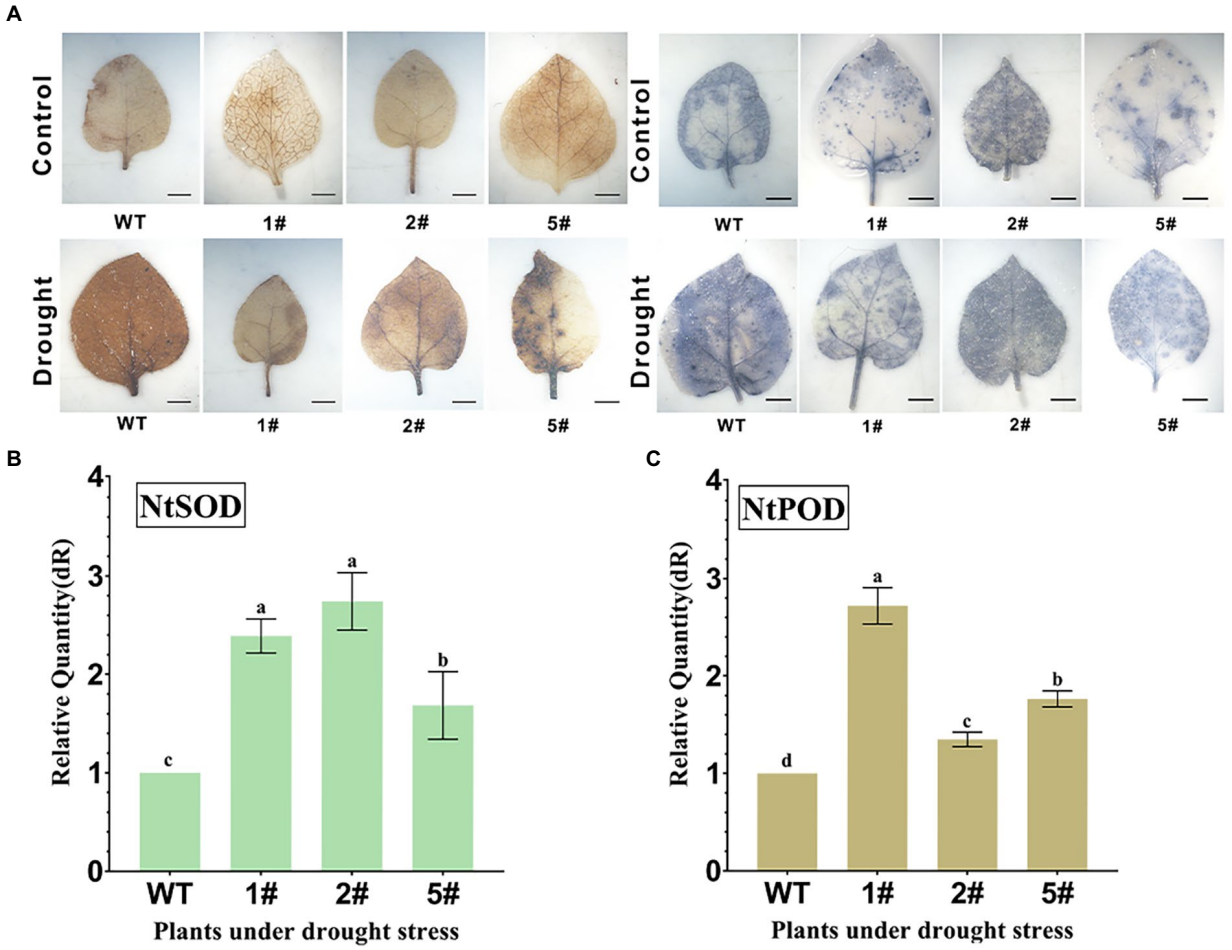
**(A)** Analysis of H_2_O_2_ and superoxide anion in tobacco leaves under drought conditions. **(B)** Quantitative expression of *NtSOD* in real time under drought stress. **(C)** *NtPOD* quantitative expression in real time under drought stress. Error lines indicate standard errors of three biological replicates with significant differences at the *p* < 0.05 level. Bar, 2 mm.

Wild-type and transgenic tobacco seedlings cultured in soil for 20 days were subjected to natural drought treatment for 15 days, and drought-treated and untreated tobacco leaves were cut and subjected to DAB staining and NBT staining, respectively (Figure 7A). DAB staining was deepened in drought-treated leaves, but lighter in transgenic leaves compared to wild type. NBT staining was similar to DAB staining, indicating that the scavenging ability of overexpressed tobacco was stronger for superoxide anion than wild type. This may indicate that *AfNAC1* overexpressing tobacco improves plant drought tolerance by scavenging ROS. By qRT-PCR analysis, the gene expression of *NtSOD* and *NtPOD* in overexpressing tobacco was higher than that in wild-type tobacco under drought stress (Figures 7B,C). In addition, the physiological and biochemical indicators of tobacco were also measured, and the results showed that the chlorophyll, proline, SOD, and POD in the overexpressed tobacco were higher than those in the wild type (Supplementary Figure S4).

**Figure 8:**
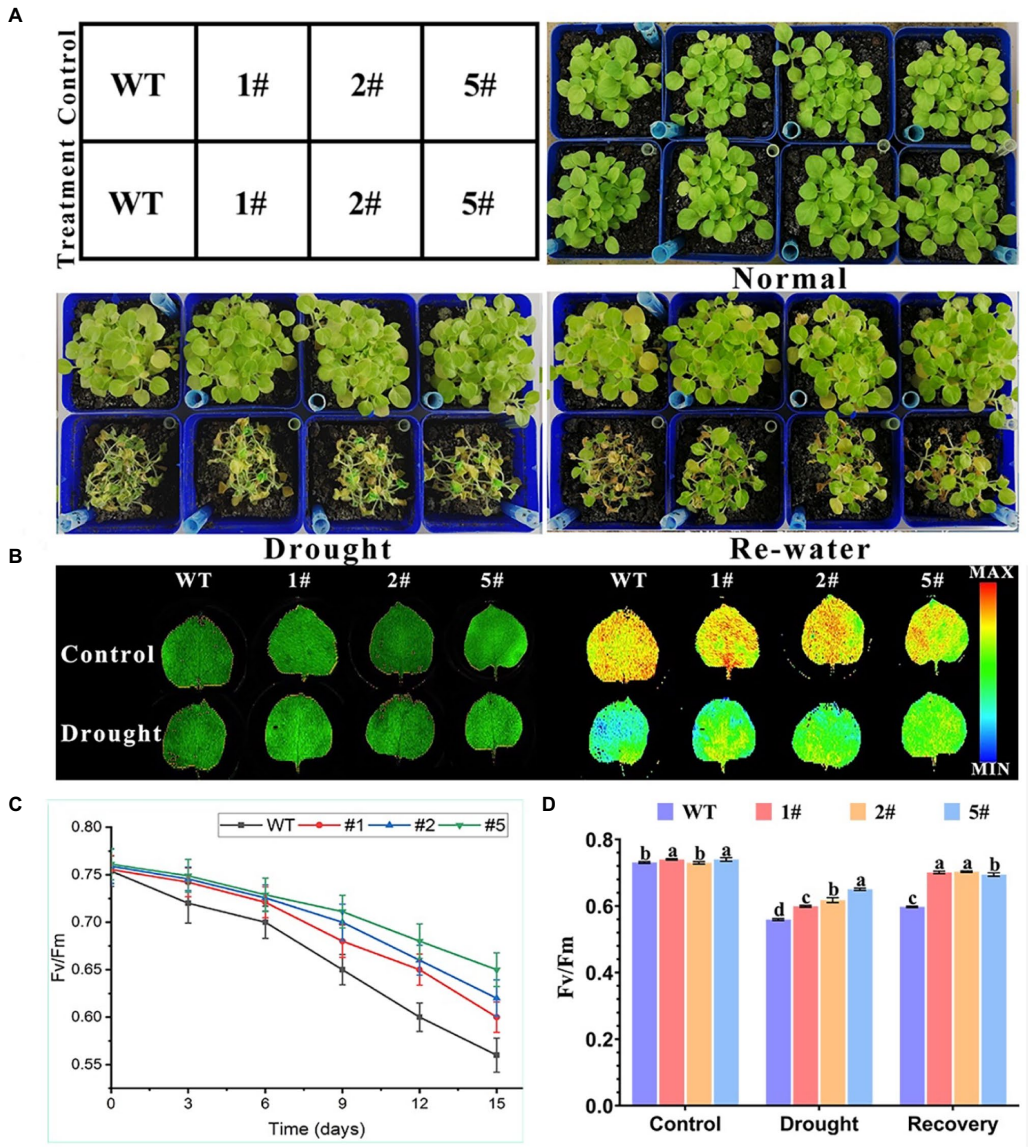
Overexpression of *AfNAC1* improves the resistance of tobacco to drought stress. **(A)** Tobacco phenotypes of transgenic tobacco before drought treatment, under drought treatment, and after re-watering treatment. **(B)** RGB image on the left, chlorophyll fluorescence parameter image on the right. **(C)** Fv/Fm values of a strain overexpressing *AfNAC1* and wild-type plants at days 0, 3, 6, 9, 12, and 15 after watering was stopped. **(D)** Control, drought treatment, and 3 days after watering were resumed. Fv/Fm values of wild-type tobacco WT compared with those of tobacco overexpressing *AfNAC1*; Error lines indicate standard errors of three biological replicates with significant differences at the *p* < 0.05 level.

Wild-type tobacco and transgenic tobacco is sown in soil for 20 days and were taken to control water and drought treatment. The phenotypic changes were investigated after 15 days and 3 days of rehydration. The Fv/Fm values of tobacco during the drought treatment period and after rehydration were detected by instrument value (Figure 8). After 15 days of water-controlled natural drought treatment, tobacco leaves were wilted and yellow, and transgenic tobacco still had bright green leaves. After 3 days of rehydration, transgenic tobacco revived significantly more leaves than WT; PSII maximum photochemical efficiency determined by FluorCam open chlorophyll fluorescence imaging system (Fv/Fm) values at 15 days of drought stress showed that WT was significantly lower than the three transgenic lines. After rehydration, the Fv/Fm values of transgenic tobacco were close to the level of untreated control, and that of WT was still very low. A comparison of chlorophyll fluorescence imaging of wild-type and transgenic tobacco showed that before drought treatment, chlorophyll fluorescence imaging of all plants tended to be bright orange-yellow regions; after drought treatment, wild-type tobacco had larger dark blue low-dark regions than overexpressed tobacco. These results suggest that *AfNAC1* overexpressing tobacco can restore photosynthesis better than wild type under drought rehydration treatment and improve drought tolerance.

## Discussion and conclusion

Water deficit (drought) is one of the most detrimental factors affecting plant growth and development, and NAC transcription factors play a key role in different developmental and stress responses of plants. They play an important role in regulating plant growth and development, affecting cell wall formation and coping with abiotic stresses (Meng et al., 2007). AfNAC1 is a hydrophilic protein encoding a specific NAM structural domain. 20% PEG stress upregulates the relative expression of *AfNAC1* and the stress time gradient shows a gradual increase in expression in leaves and an increase followed by a decrease in expression in roots. Transcriptional regulators mostly function in the nucleus by binding to downstream target genes. The subcellular localization of AfNAC1 protein in this study suggests that it is a nuclear-localized protein. It has been shown that ChNAC4 protein is a cell membrane protein that exhibits activation properties in the nucleus and forms homodimers that are regulated in response to salt and drought (Trishla and Kirti, 2021). Many NAC TFs have also been reported to enhance tolerance to biotic stresses. Overexpression of Arabidopsis *ANAC019*, *ANAC055,* and *ANAC072* has been shown to confer drought tolerance in plants (Fujita et al., 2004). Figueroa et al. (2021) concluded from the meta-analysis that driving NAC expression using the *CaMV35S* strong promoter improved plant drought tolerance significantly more than other promoters. Li et al. (2021) found that overexpression of *VyDOF8* in tobacco significantly enhanced drought tolerance under drought conditions, with better root length and lateral root number than wild-type tobacco. Lang (2021) found that *XsNAC* genes have different expression profiles in response to various abiotic stresses through transcriptome analysis, suggesting that *XsNAC* genes This suggests that *XsNAC* genes may also play a key role in the response to drought stress. The results showed that the malondialdehyde content of transgenic tobacco was significantly lower than that of wild-type tobacco under drought conditions, and the root length of transgenic tobacco was also longer than that of wild-type tobacco. Under stress conditions, the plants were able to activate their antioxidant enzyme protection system, thus mitigating the damage caused by reactive oxygen species and eventually scavenging a large amount of ROS accumulated in the plants (Chen et al., 2016). The experimental data showed that the transgenic plants mitigated the oxidative damage caused by drought stress and enhanced the drought resistance of the transgenic plants, which is like the results of Li et al. NAC proteins are transcription factors with plant specificity. Vikas Shalibhadra Trishla et al. studied *GhNAC4* and found that its transfer into tobacco resulted in higher seed germination and longer root length in transgenic tobacco than in wild-type tobacco, indicating that *GhNAC4* is involved in the process of plant response to drought stress, which is consistent with the results of the present experiment. In this study, to analyze the drought resistance of the *AfNAC1* gene, we detected the content of H_2_O_2_ and superoxide anion in leaves by DAB staining and NBT staining. The expression levels of *NtSOD* and *NtPOD* genes were upregulated after drought treatment, and we hypothesized that overexpression of *AfNAC1* could reduce the accumulation of ROS and enhance the drought resistance of plants. Drought resistance was further demonstrated by measuring chlorophyll, proline, and antioxidant enzyme contents of wild-type and transgenic tobacco after natural drought. In addition, drought stress affected the photosynthesis of plants and altered chlorophyll fluorescence parameters. In this study, the Fv/Fm values of transgenic tobacco were found to be close to those of the untreated control after rehydration recovery, whereas the Fv/Fm values of WT remained low. This indicates that *AfNAC1* improved the resistance of tobacco to drought stress, reflecting the important role of stress resistance through plant photosynthetic responses; the use of *AfNAC1* provides a reference for improving plant drought resistance.

## Data availability statement

The datasets presented in this study can be found in online repositories. The names of the repository/repositories and accession number(s) can be found in the article/Supplementary material.

## Author contributions

ML: writing—original draft and visualization. ZL: visualization. CL: writing—review and editing. KW: writing—review and editing. FZ: writing—review and editing. ZW, XL, and QG: conceptualization, funding acquisition, project administration, and writing—review and editing. All authors contributed to the article and approved the submitted version.

## Funding

This work is supported by the National Natural Science Foundation of China (32171989 and 31870300); Fundamental Research Funds for the Central Universities (2572021DS03); and Heilongjiang Touyan Innovation Team Program (Tree Genetics and Breeding Innovation Team).

## Conflict of interest

The authors declare that the research was conducted in the absence of any commercial or financial relationships that could be construed as a potential conflict of interest.

## Publisher’s note

All claims expressed in this article are solely those of the authors and do not necessarily represent those of their affiliated organizations, or those of the publisher, the editors and the reviewers. Any product that may be evaluated in this article, or claim that may be made by its manufacturer, is not guaranteed or endorsed by the publisher.

## Footnotes

^1^ https://bigd.big.ac.cn/gsub/

^2^ http://smart.embl-heidelberg.de/smart/set_mode.cgi?NORMAL=1
