# Supplementary material for: Drought resistance of tobacco overexpressing the AfNAC1 gene of Amorpha fruticosa Linn
Source: Front Plant Sci. 2022 Aug 16;13:980171. doi: 10.3389/fpls.2022.980171 (PMC9425102; doi:10.3389/fpls.2022.980171)
Supplement: Supplementary file 1 [file Data_Sheet_1.docx]

Supplementary Material

# Supplementary Tables

Table1.Primers needed in the experiment

| Name | 5'- -3' sequence |
| --- | --- |
| *AfNAC1.F1* | *ATGGATGGCAAAACAAGCTC* |
| *AfNAC1.R2* | *TCATTTCTTGTTCACCGTCAT* |
| *qAfNAC1.F3* | *CCAACGCAGGGTATGGAGAA* |
| *qAfNAC1.R4* | *TGTCTGTGCCAGTAGCCTTC* |
| *AfActin1-F* | *ACAAGGCGGTTAAGGTTGGT* |
| *AfActin1-R* | *GTTCTGGGCTTGGTTCCCTT* |
| *AfNAC1.F5* | *TCTAGATTATGGATGGCAAAACAAG* |
| *AfNAC1.R6* | *GTCGACATTTCTTGTTCACCGTCAT* |
| *AfNAC1.F7* | *GGATCCATGGATGGCAAAACAAG* |
| *AfNAC1.R8* | *CTCGAGTCATTTCTTGTTCACCGTCAT* |
| PBI121-FW | *TCATTTCATTTGGAGAGAACAC* |
| PBI121-RV | *TTGCCAAATGTTTGAACGATC* |
| *NtActin-F* | *CTGCTGGAATTCACGAAACA* |
| *NtActin-R* | *GCCACCACCTTGATCTTCAT* |
| *NtSOD-F* | *GTGAGCAGACGGACCTTAGC* |
| *NtSOD-R* | *GGCGTCATGTAGCTGTTCAA* |
| *NtPOD-F* | *TCCTGGAGTTGTTGCCTTGCTAG* |
| *NtPOD-R* | *GCTTAGTCCTCTTGTCCGTTGCTA* |

# Supplementary Figures

**
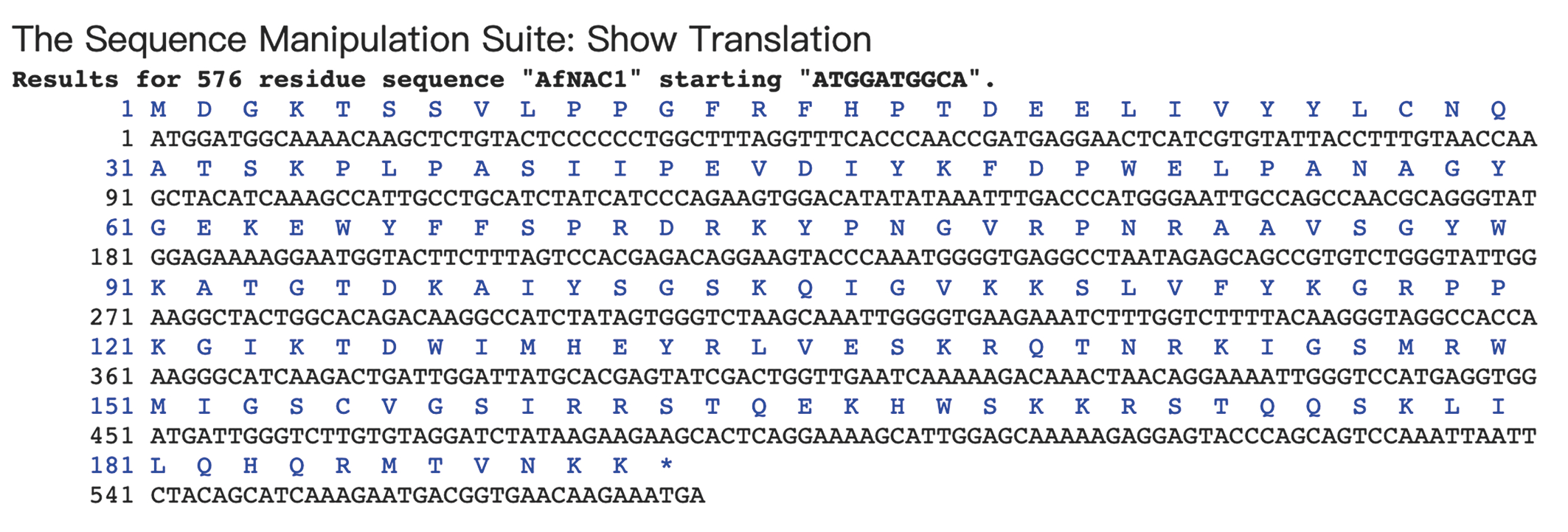
**

**Supplementary Figure 1.** The ORF of *AfNAC1* is 576bp, encoding 191 amino acids.


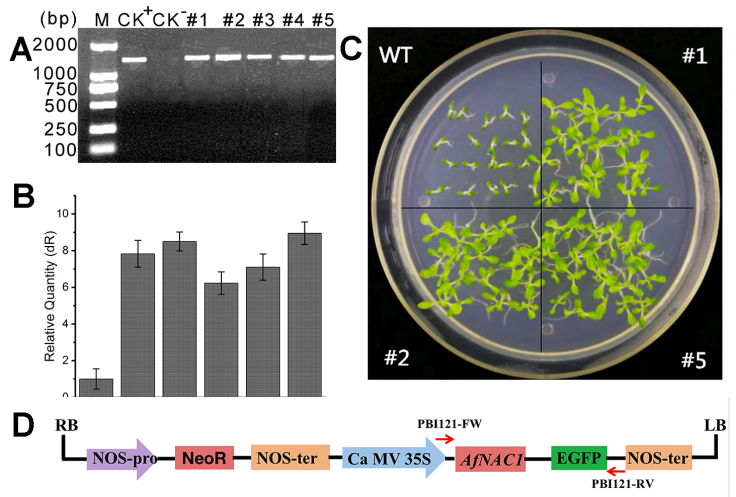


**Supplementary Figure 2.** (**A**) PCR identification of transgenic plants using the PBI121 universal primer. (**B**) Relative expression analysis of transgenic plants. (**C**) Screening of transgenic plants and wild-type plants for resistance under Kana treatment. (**D**)Diagram of PBI121-*AfNAC1*-*GFP.*


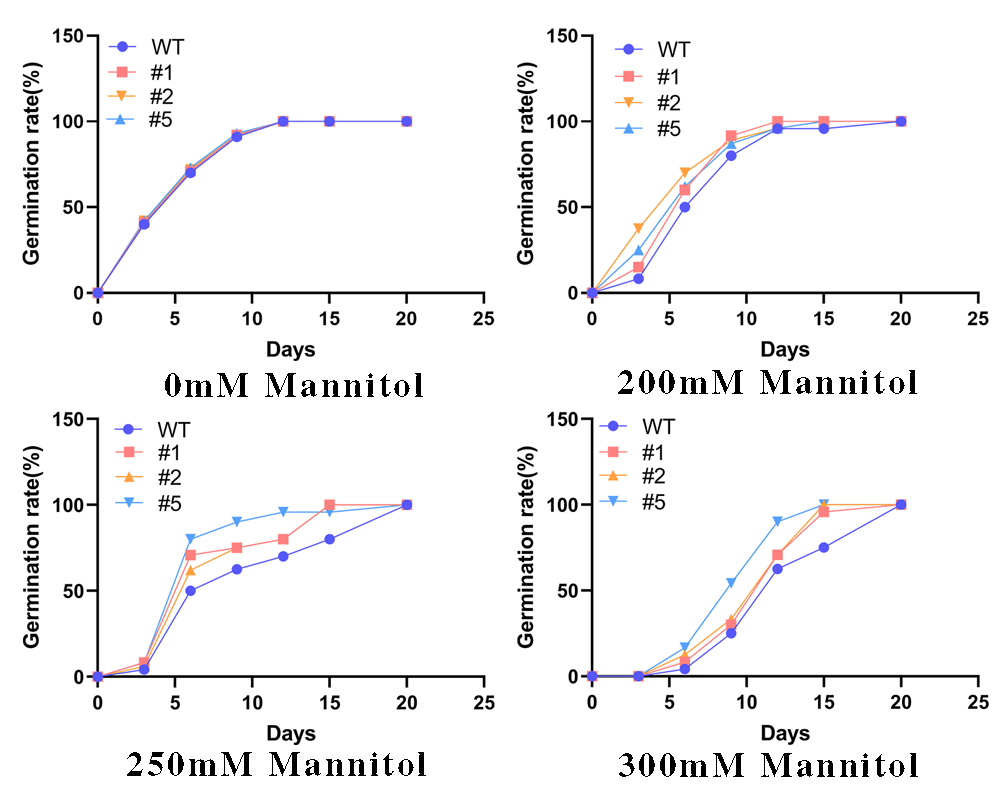


**Supplementary Figure 3.** Germination rates of AfNAC1 overexpression lines and wild-type plants under 0 mM, 200 mM, 250 mM and 300 mM mannitol stress.


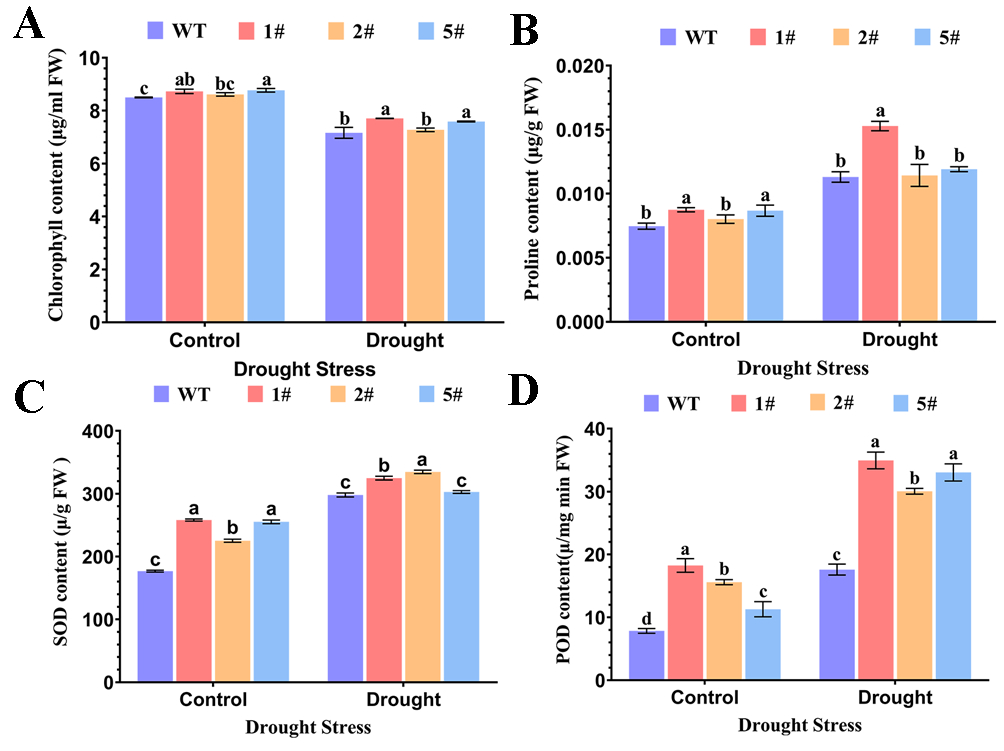


**Supplementary Figure 4.** (A) Chlorophyll content determination. (B) Proline content determination. (C) SOD content determination. (D) POD content determination.
